# Early ketamine exposure results in cardiac enlargement and heart dysfunction in *Xenopus* embryos

**DOI:** 10.1186/s12871-016-0188-z

**Published:** 2016-04-18

**Authors:** Ran Guo, Guangjian Liu, Min Du, Yu Shi, Pu Jiang, Xiaoli Liu, Lan Liu, Jianxia Liu, Ying Xu

**Affiliations:** 1 Department of Anesthesiology, Children’s Hospital of Chongqing Medical University, Ministry of Education Key Laboratory of Child Development and Disorders, Key Laboratory of Pediatrics in Chongqing, Chongqing International Science and Technology Cooperation Center for Child Development and Disorders, Chongqing Key Laboratory of Translational Medical Research in Cognitive Development and Learning and Memory Disorders, Chongqing, 400014 PR China; 2 Department of Anesthesiology, Children’s Hospital of Chongqing Medical University, No.136, Zhongshan Er Lu, Yuzhong District, Chongqing City, 400014 PR China; 3 Department of Clinical laboratory, Children’s Hospital of Chongqing Medical University, Chongqing, 400014 PR China; 4 Department of Forensic Medicine, College of Basic Medicine, Chongqing Medical University, Chongqing, 400016 PR China; 5 Department of Pulmonary and Critical Care Medicine, Brigham and Women’s Hospital, Boston, MA 02115 USA

**Keywords:** Ketamine, Drug exposure, *XMLC2*, *Xenopus laevis*, Heart development

## Abstract

**Background:**

Ketamine is a commonly used clinical anesthetic and a popular recreational drug. However, with the exception of studies about the nervous system, studies about the effect of early ketamine exposure on embryos are rare. *Xenopus laevis* is a commonly used vertebrate model for assessing teratogenicity. Therefore, we treated *Xenopus* embryos with ketamine to evaluate its teratogenicity on embryos.

**Methods:**

*Xenopus* embryos were treated with ketamine from stages 8 to 21. Embryonic and cardiac morphology were analyzed using living embryo imaging and whole-mount RNA *in situ* hybridization (WMISH). Heart function was measured by heart rate and ventricular shortening fraction (VSF). The mRNA expression levels of several heart development-related genes were determined by reverse transcription quantitative polymerase chain reaction (RT-qPCR). The protein expression levels of XMLC2, phospho-histone H3 (pH3) and histone H3 were determined by western blot.

**Results:**

Ketamine caused concentration-dependent increases in mortality and shortening of body length. At a dose of 0.5 mg/ml, ketamine exposure resulted in cardiac enlargement as the primary manifestation of several malformations: gut defects, a curved axis and shortened body length. Cardiac cells underwent increased proliferation. Moreover, the heart rate and ventricular shortening fraction were decreased, findings indicative of heart dysfunction. XMLC2 expression levels were down-regulated at stages 28, 32/33, 35/36 and 46.

**Conclusions:**

Ketamine exposure during early development has teratogenic effects on *Xenopus* embryos. The heart enlargement and decreased VSF may result from the down-regulation of XMLC2 mRNA and protein levels. These findings provide new insight into the potential fetal defects induced by ketamine exposure during early pregnancy.

**Electronic supplementary material:**

The online version of this article (doi:10.1186/s12871-016-0188-z) contains supplementary material, which is available to authorized users.

## Background

Ketamine, a non-competitive inhibitor of N-methyl-D-aspartate (NMDA) receptors, is widely used for sedation, analgesia, and the induction and maintenance of anesthesia in humans, particularly in pediatric patients [1, 2]. Moreover, as a hallucinogenic agent, ketamine is increasingly used for non-medical purposes and is one of the most popular “club drugs” [3]. Epidemiological data indicate that 30 % of ketamine abusers are female [4] and are often less than 27 years of age [5], which is childbearing age. Therefore, it is of great significance to study the effect of ketamine exposure during pregnancy on embryonic development. However, clinical data regarding its effect on fetuses are rare. Previous studies using animal models to investigate the effects of ketamine on embryogenesis have focused primarily on neurological issues during either the late embryonic period or the neonatal period [6–9]. Herein, we examined the impact of ketamine exposure during early embryonic development.

*Xenopus* is a commonly used vertebrate model for studying embryonic development mechanisms and drug teratogenicity [10]. It produces massive eggs, and the transparency of tadpoles facilitates the detection of internal organization defects. More importantly, *Xenopus* is similar to humans in terms of organ development, physiology and gene expression [11]. Therefore, we used *Xenopus* to investigate the teratogenic effects of early ketamine exposure on embryos in this study.

## Methods

### Animals

Studies with *Xenopus laevis* were approved by the local ethics committee (Chongqing Medical University, Chongqing, China). *X. laevis* were maintained in a recirculating system at 21 ± 1 °C with a 12-h light–dark cycle. Fertilization was stimulated by the administration of human chorionic gonadotropin (HCG).

### Embryo treatments

Fertilized oocytes were washed in 2 % L-Cysteine (pH 8.0) to remove the jelly coat. *Xenopus laevis* embryos were collected and staged according to Nieuwkoop and Faber [12].

Experiments were conducted in 0.3× Modified Barth’s Saline (MBS, 88 mM NaCl, 1 mM KCl, 0.7 mM CaCl2, 1 mM MgSO4, 2.5 mM NaHCO3, and 5 mM HEPES, pH 7.8). Working solutions of ketamine (Sigma, St Louis, MO, USA; cat.no.K2753) were dissolved in 0.3 × MBS, and the treatment was administered at various concentrations from stage 8 (before the gastrula stage) to stage 21 (complete neural tube closure). After treatment, the embryos were transferred to 0.3× MBS and cultured in six-well plates at 21 °C; each well contained 10 embryos in 5 ml of 0.3× MBS. The solution and drug were changed every 24 h. Each treatment group was tested 3 separate times.

### Live embryo imaging

After inducing anesthesia with tricaine mesylate for 1 min, the embryos at stage 46 were immediately recorded using a stereo fluorescence microscope Stereo Lumar V12 (Zeiss, Germany) and photographed in diastole upon reaching the end diastolic volume using an SMZ 1500 (Nikon, Japan) at 10× magnification for the whole embryos and at 60× magnification for the heart region. Embryo body lengths were measured three times using Image J software (ver 1.47, Bethesda, MD), and the averages were analyzed.

### Heart rate and ventricular contractility analysis

After inducing anesthesia for 1 min, the embryos at stage 46 were randomly selected for heart rate counts. Ventricular beats were counted for 1 min. At least three measurements were obtained, and the average was used for statistical analysis.

Cardiac contractions were recorded with Stereo Lumar V12. The lengths of ventricles in diastolic and systolic conditions were measured to calculate the ventricular shortening fraction (VSF) [13, 14]: VSF = (ventricular length at diastole–ventricular length at systole)/ventricular length at diastole.

### Whole-mount RNA in situ hybridization (WMISH)

Digoxigenin (DIG)-labeled antisense RNA probes for *XNkx2.5*, *XTnIc* and *XMLC2* were constructed based on previous research [15], and WMISH was performed as previously described [16]. Embryos were imaged using a Nikon SMZ 1500 stereomicroscope at 60× magnification for the heart region.

### RNA isolation and reverse transcription quantitative polymerase chain reaction (RT-qPCR)

Total RNA was extracted from ten whole embryos or hearts per experimental condition using TRIzol Reagent (Invitrogen, Grand Island, NY) according to the manufacturer’s instructions. cDNA synthesis was conducted using a PrimeScript RT Reagent Kit (Takara, Otsu, Shiga, Japan). RT-qPCR was performed in triplicate using 10-fold diluted cDNA and a SYBR Green I kit (Tiangen, Beijing, China) on a CFX96 real-time PCR machine. The following primers were used for RT-qPCR:

*XNkx2.5*: forward 5′-GGC TAC CAC CTC CAA GAC G-3′, reverse 5′-GTT GGA GTG GGC AGG GTA AG-3′;

*XTnIc*: forward 5′-TGA ATC CAC TGG GGC TGT TG-3′, reverse 5′-AGA GTA ACG GCC TTC GAA CA-3′;

*MHC*-*alpha*: forward 5′-TCA AGG CTG GTT TGT TGG GT-3′, reverse 5′-AAC CAG AAG GGC ATC TCT GC-3′;

*XMLC2*: forward 5′-GCG CAA TGG TCT TGC TCT TC-3′, reverse 5′-GAG ATC GTG GAG GGC AAA GT-3′;

*GAPDH*: forward 5′-TAG TTG GCG TGA ACC ATG AG-3′, reverse 5′-GCC AAA GTT GTC GTT GAT GA-3′.

The primers for *gata4* and *gata6b* were used as described [15].

The PCR cycling conditions were as follows: 1 cycle at 95 °C for 3 min, 39 cycles at 95 °C for 10 s and 60 °C for 30 s. GAPDH mRNA was used as an endogenous control. The relative quantification was calculated using the 2^-△△ct^ method.

### Western blot analysis

Protein was extracted from ten whole embryos or 200 embryonic hearts per experimental condition, and western blotting was performed using standard procedures. Monoclonal mouse anti-human MLC2 from Novus (Littleton, CO; cat. no. NBP1-30249), polyclonal rabbit anti-human histone H3 from proteintech (Chicago, IL; cat. no. 17168-1-AP) and polyclonal rabbit anti-rat GAPDH from EnoGene (Nanjing, Jiangsu, China; cat. no.E12-052) were diluted by 1:2000. Polyclonal rabbit anti-human pH3 from Santa Cruz Biotechnology (Dallas, Texas; cat. no.sc-8656-R) was diluted by 1:500. GAPDH was applied as an internal reference. Immunoreactive bands were quantified by densitometry using ImageJ.

### Statistical analysis

SPSS Statistics version 17.0 (Chicago, IL, USA) was used for statistical analysis. Comparisons among different concentration groups were made by one-way ANOVA. Comparisons between two groups were conducted using either unpaired t-tests or chi-square tests for different data. *P*-values <0.05 were considered statistically significant.

All experiments were repeated at least 3 times.

## Results

### Ketamine induces embryonic malformations

**Fig. 1:**
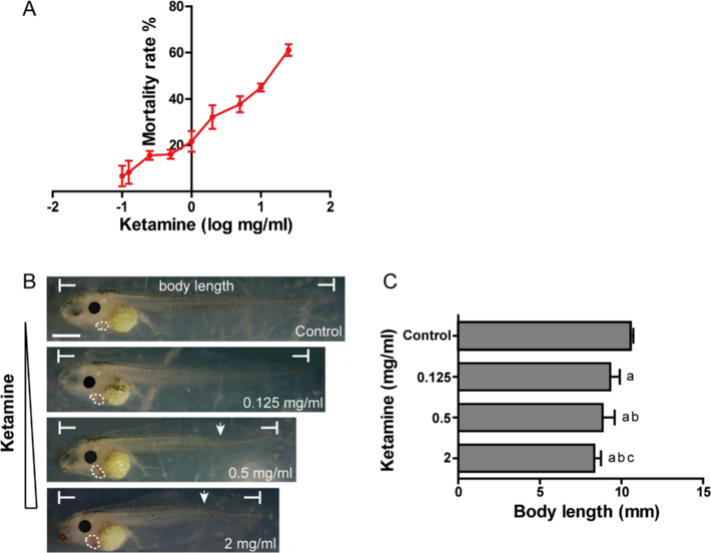
The effect of ketamine on embryos at stage 46. **a** Mortality rate of increasing concentrations of ketamine (0.1, 0.125, 0.25, 0.5, 1, 2, 5, 10, 25 mg/ml). **b** Malformations at three exposure concentrations of ketamine (0.125, 0.5, 2 mg/ml). Hearts are marked by *white punctiform curves*. The *arrowhead* indicates a curved axis. *Scale bar* = 100 μm. **c** Comparison of body lengths at three exposure concentrations. ^a^ *P* < 0.05 vs. control group; ^b^ *P* < 0.05 vs. 0.125 mg/ml ketamine-treated group; ^c^ *P* < 0.05 vs. 0.5 mg/ml ketamine-treated group. The data represent the means ± SD

*Xenopus* embryos were exposed to ketamine from stage 8 to stage 21. When the embryos developed to stage 46, we counted embryonic mortality within each ketamine-treated group. The result demonstrated that as the exposure concentration increased, the mortality rate also increased (Fig. 1a). Ketamine concentrations of 0.125, 0.5 and 2 mg/ml were chosen to observe the embryonic phenotype. It demonstrated that with increasing ketamine concentrations, body lengths gradually shortened (*P* < 0.05), hearts enlarged, and curved body axis was found at high concentrations (Fig. 1b-c).

The numbers of deaths and malformations in the three ketamine-treated groups were analyzed (Table 1). It demonstrated that after exposure to 0.5 mg/ml ketamine, the embryonic malformation rate was significantly increased compared with the specimens exposed to 0.125 mg/ml (*P* < 0.05), whereas the death rate was significantly decreased compared with the specimens exposed to 2 mg/ml (*P* < 0.05). Moreover, a ketamine concentration of 0.5 mg/ml did not affect the embryonic death rate (*P* > 0.05). Thus, we chose this concentration for subsequent experiments.

**Table 1:** The effect of ketamine at three concentrations on embryonic death and malformation

	Control	Ketamine (mg/ml)		
		0.125	0.5	2
Death	17 (9.44 %)	16 (8.89 %)	29^b^ (16.11 %)	52^a,c^ (28.89 %)
Malformation	4 (2.45 %)	117^a^ (71.34 %)	129^a,b^ (85.43 %)	108^a^ (84.38 %)
Total	180	180	180	180

^a^ *P* < 0.05 vs. control group; ^b^ *P* < 0.05 vs. 0.125 mg/ml ketamine-treated group; ^c^ *P* < 0.05 vs. 0.5 mg/ml ketamine-treated group

### Ketamine predominantly causes cardiac enlargement

**Fig. 2:**
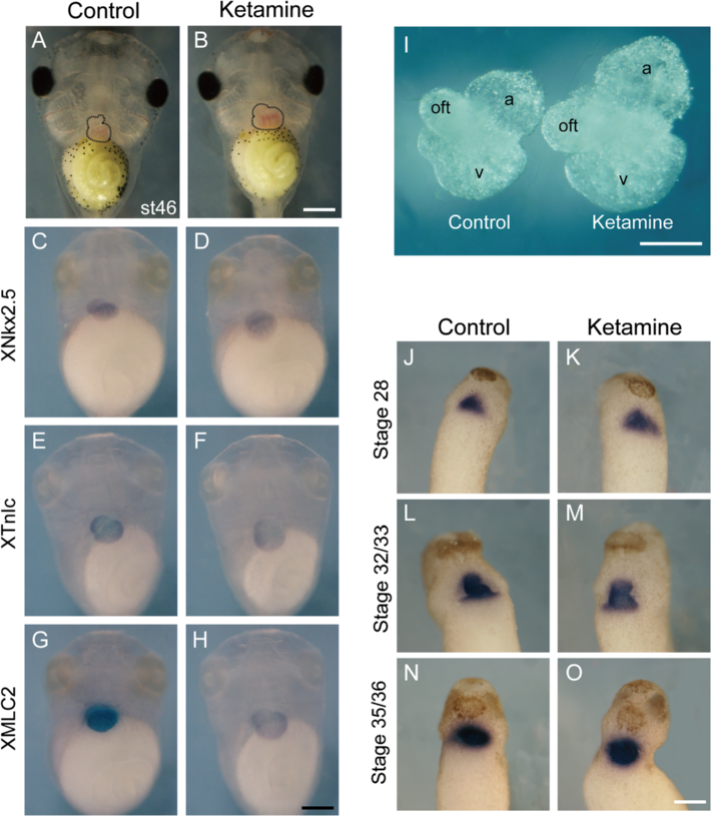
The effect of ketamine on cardiac morphology. **a**-**h** Embryos at stage 46. **a**-**b** Live embryos. **c**-**h** Whole-mount RNA *in situ* hybridization (WMISH) analysis of the heart-specific gene *XNkx2.5* and myocardium-specific genes *XTnIc* and *XMLC2*. **i** The dissected hearts from embryos at stage 46. *oft*, outflow tract; *a*, atria; *v*, ventricle. **j**-**o** WMISH analysis of *XMLC2* at stage 28, 32/33 and 35/36. *Scale bar* = 20 μm

After exposure to 0.5 mg/ml ketamine, no significant differences in mortality were found between the control and ketamine-treated embryos at stage 46 (Table 2). However, it caused heart defects (82.12 %), gut defects (4.64 %), a curved axis (19.87 %) and a shortened body length (8.86 ± 0.86 mm). Therefore, heart defects were the most commonly observed phenotype in the ketamine-treated embryos compared with the controls (82.12 versus 1.84 %), and heart enlargement (76.82 %) was the primary malformation (Table 2). The heart size of the ketamine-treated embryos was increased compared with the control embryos as determined via living embryo imaging, WMISH for the heart and myocardium, and the dissection of the heart tissues at four stages (stage 28, 32/33, 35/36 and 46) from heart tube formation to heart maturity (Fig. 2).

**Table 2:** Characteristics of ketamine-treated embryos (0.5 mg/ml) at stage 46

Characteristic	Control	Ketamine (0.5 mg/ml)
Mortality	17 (9.44 %)	29 (16.11 %)
Heart defects	3 (1.84 %)	124* (82.12 %)
Enlarged	2 (1.23 %)	116* (76.82 %)
Undefined	1 (0.61 %)	8* (5.30 %)
Gut defects	1 (0.61 %)	7* (4.64 %)
Curved axis	3 (1.84 %)	30* (19.87 %)
Small head	0 (0.00)	1 (0.66 %)
Body length (mm)	10.53 ± 0.20	8.86 ± 0.86*
Total embryos	180	180

Note that one embryo can have various malformations. All malformations were individually counted and analyzed. The body length values are expressed as the means ± SD. *P < 0.05

Because of the remarkable heart defects, we subsequently investigated the specific effect of ketamine on heart during embryonic development.

### Ketamine increases cardiac cell proliferation

**Fig. 3:**
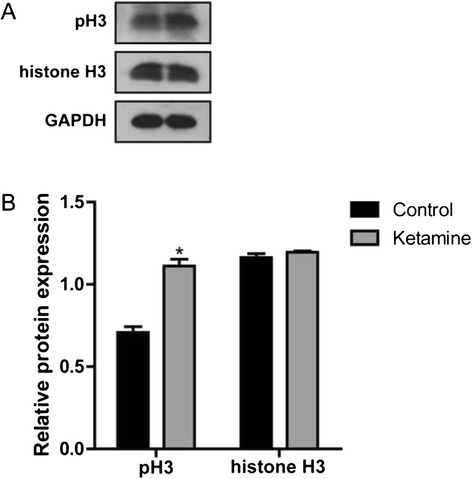
The effect of ketamine on cardiac cell proliferation. **a** Phospho-histone H3 (pH3) and histone H3 protein levels. **b** Quantitation of the levels of pH3 and histone H3 protein shown in (**a**). The data are expressed as the means ± SD

We dissected out the heart tissues to detect cell proliferation using western blot. The mitosis marker phospho-histone H3 (pH3) was used as a marker of cell proliferation. The results showed that after ketamine exposure, pH3 expression level was significantly up-regulated (*P* < 0.05), while histone H3 expression level showed no significant difference in those two groups (Fig. 3), suggesting that ketamine increased cardiac cell proliferation.

### Ketamine results in embryonic heart dysfunction

**Fig. 4:**
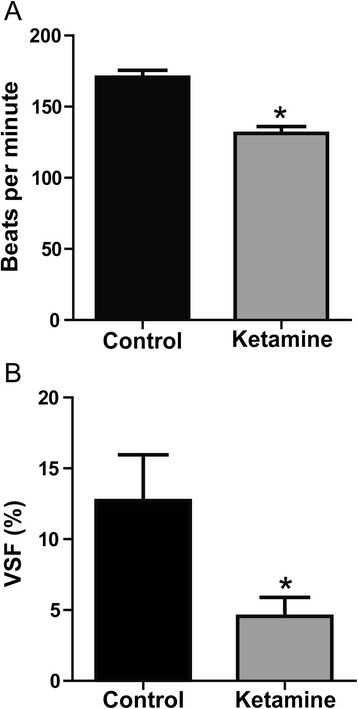
The effect of ketamine on heart function. **a** Comparison of the heart rate (beats per minute) in the control and ketamine-treated embryos. *n* = 10. *P < 0.05. **b** Comparison of ventricular shortening fraction (VSF) in the control and ketamine-treated embryos. *n* = 6. *P < 0.05. The data are expressed as the means ± SD

To investigate the effect of ketamine exposure on cardiac function, we determined the embryonic heart rate and recorded cardiac impulse to calculate the VSF via stereo microscopy at stage 46 (see Additional files 1 and 2). The results demonstrated that the heart rates in the control and ketamine-treated groups were 169.96 ± 5.25 bpm and 130.82 ± 4.87 bpm, respectively (Fig. 4a), and the VSFs in the two groups were (12.72 ± 3.25)% and (4.56 ± 1.33)%, respectively (Fig. 4b). Thus, both the heart rate and VSF in the ketamine-treated group were significantly decreased compared with the control group, indicating that ketamine exposure affects embryonic heart function.

### Ketamine down-regulates XMLC2 expression

**Fig. 5:**
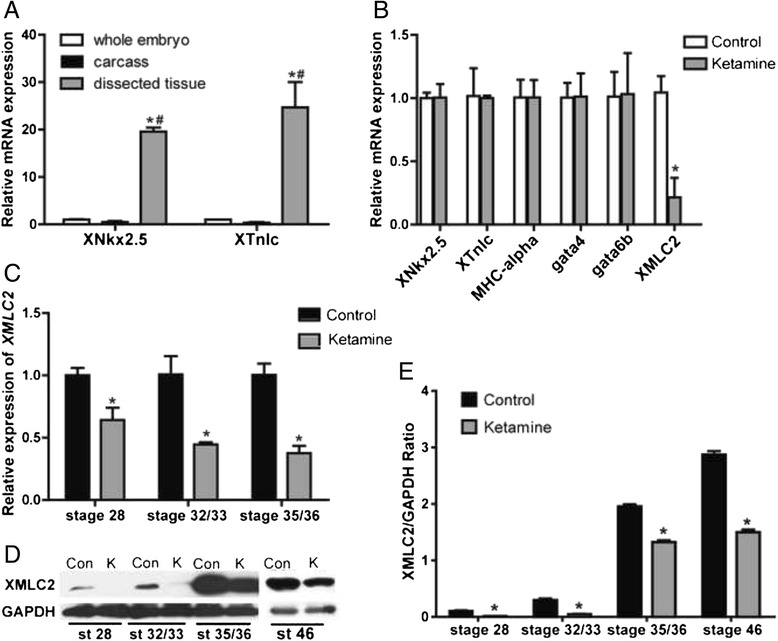
The effect of ketamine on heart development-related gene expression. **a** The mRNA levels of heart-specific genes *XNkx2.5* and *XTnIc* in whole embryos, carcasses and dissected tissues at stage 46. *P < 0.05, #*P* < 0.05. **b** The mRNA levels of heart development-related genes, *XNkx2.5*, *XTnIc*, *MHC*-*alpha*, *gata4*, *gata6b* and *XMLC2* in dissected tissues at stage 46. *P < 0.05. **c** XMLC2 mRNA levels at stage 28, 32/33 and 35/36. **d** XMLC2 protein levels at stage 28, 32/33, 35/36 and 46. **e** Quantitation of the levels of XMLC2 protein shown in D. The data are expressed as the means ± SD. *Con* control, *K* ketamine, *st* stage

We firstly anesthetized the embryos at stage 46 and dissected out the heart regions under a microscope. The dissected tissues were subsequently used to detect the mRNA expression levels of several heart development-related genes using RT-qPCR. In the dissected tissues, the expression levels of *XNkx2.5* and *XTnIc* were significantly higher than that in the whole embryos and the remaining carcasses (*P* < 0.05) (Fig. 5a), confirming that the dissected tissues were hearts. Then, we examined the mRNA expression levels of *XNkx2.5*, *XTnIc*, *MHC*-*alpha*, *gata4*, *gata6b* and *XMLC2* in these tissues (Fig. 5b). The data indicated that only XMLC2 mRNA was decreased in the ketamine-treated group (*P* < 0.05), whereas the other gene expression levels showed no significant difference in the two groups. Furthermore, we examined XMLC2 mRNA expression during three early stages of heart development (Fig. 5c). The expression levels were all significantly decreased in the ketamine-treated group (*P* < 0.05), which were consistent with the results observed at stage 46. We then detected XMLC2 protein expression using western blot at four stages. The result demonstrated that the expression levels were all down-regulated in the ketamine-treated groups (Fig. 5d-e).

## Discussion

We studied cardiac morphology, cardiac function and the expression of genes associated with heart development after ketamine exposure primarily at stage 46. Because at this stage, the *Xenopus* embryonic heart has matured [17]. Furthermore, there are three key stages for heart development: stage 28, heart tube formation; stage 32/33, contractile myocardial tube formation and looping initiation; and stage 35/36, obvious heart looping [18, 19]. Therefore, we chose these three stages to investigate the effect of ketamine exposure on early embryonic cardiomyogenesis.

Thus far, reports on the effects of early embryonic exposure to ketamine on organs or systems other than the nervous system are rare. An in vivo research demonstrated that ketamine exposure during the early development of zebrafish embryos caused concentration-dependent increases in anomalies and mortality; the most prominent deformities were enlarged organs and tail/spine anomalies [20], which is consistent with the results of our study. Additionally, we found that the shortening of embryonic body length is also in concentration-dependent manners.

At a dose of 0.5 mg/ml, the ketamine-treated embryos exhibited several malformations. However, the most prominent phenotype was an enlarged heart. Moreover, the cardiac cells underwent significantly increased proliferation. Our results also showed that at each detected stage of heart development, from heart tube formation to heart maturity, XMLC2 mRNA and protein expression levels were significantly down-regulated after ketamine exposure. It has been reported that MLC2, myosin regulatory light chain 2, is essential for normal myocardium development [21]. A defect of *MLC2* gene results in myocardial malformations [21], and missense mutations of *MLC2* in humans lead to hypertrophic cardiomyopathy [22, 23], resulting in heart enlargement and decreased cardiac contractility. In addition, deletion of MLC2 in mice results in cardiac enlargement [24]. Therefore, the cardiac enlargement phenotype caused by ketamine may result from the down-regulation of XMLC2 mRNA and protein levels.

Furthermore, our results showed that VSF, a sensitive indicator of cardiac contractility, sharply decreased after ketamine treatment. It has been reported that MLC2 protein has a function in the maintenance of cardiac contractility. Deletion of *MLC2* causes a reduced left ventricular ejection fraction and abnormalities in myofibril assembly in embryonic mice, and results in death due to cardiac dysfunction at E12.5 [24]. Additionally, knockdown of *MLC2* in chick embryos results in poorly developed sarcomeres [25]. Because the sarcomere is the basic contractile unit, sarcomere structural abnormalities affect cardiac contractility. Therefore, the weakened cardiac contractility may be due to reduced expression levels of XMLC2 mRNA and protein.

Ketamine has been reported to reduce heart rates in pregnant and infant monkeys [26], as well as newborn human infants [27]. During zebrafish embryogenesis, ketamine exposure at both 26 and 52 h post-fertilization results in a reduced heart rate [28], which is consistent with our results. Therefore, the results of heart rate and ventricular shortening fraction demonstrate that ketamine causes embryonic cardiac dysfunction.

## Conclusions

Ketamine exposure from stage 8 to stage 21 results in concentration-dependent increases in anomalies and mortality. After 0.5 mg/ml ketamine exposure, the most prominent malformation is heart enlargement. Besides, the decrease of heart rate and VSF indicates heart dysfunction. The enlarged heart and decreased VSF may result from the down-regulation of XMLC2 mRNA and protein levels. These findings provide insight into the potential fetal defects and mechanism of ketamine exposure. However, additional studies are required to evaluate the potential risk during very early pregnancy in mammals.

## Additional files

Additional file 1: Movie of control embryo. Control embryo at stage 46, showing heart with rhythmic and strong contractions. 60× magnification. (MOV 7950 kb)

Additional file 2: Movie of ketamine-treated embryo. Note that the heart size is bigger and heart rate is slower than control embryo. The amplitude of heart beat is decreased. 60× magnification. (MOV 5408 kb)

## Abbreviations

MBS: Modified Barth’s Saline
RT-qPCR: reverse transcription quantitative polymerase chain reaction
VSF: ventricular shortening fraction
WMISH: whole-mount RNA *in situ* hybridization
